# Prevalence and Outcomes of Fears in Advanced Heart Failure: Differences Across Disease Stages

**DOI:** 10.1007/s11897-025-00705-0

**Published:** 2025-05-17

**Authors:** Mats Westas, Semyon Melnikov

**Affiliations:** 1 Department of Health, Medicine and Caring Sciences, Linköping University, Linköping, Sweden; 2 Department of Nursing Sciences, Stanley Steyer School of Health Professions, Gray Faculty of Medical and Health Sciences, Tel Aviv University, Tel Aviv, Israel

**Keywords:** Fear, Heart failure, Left-ventricular assist device, Heart transplantation

## Abstract

**Purpose of Review:**

Heart failure (HF) is a complex, multifactorial syndrome resulting from impaired heart function. When medical management of HF is ineffective, mechanical circulatory support with a left-ventricular assist device (LVAD) or heart transplantation are the only options for significantly extending patients' lives. Patients with HF experience various emotional reactions, including fears, which may impact their well-being and disease management. Understanding how fears may differentially influence patients with HF depending on the stage of the disease is thus essential for delivering personalized care.

**Recent Findings:**

Among patients with advanced HF, disease-related and existential fears were associated with anxiety, depression, sleeplessness, loss of dignity, feelings of abandonment, uncertainty about the future, and restricted physical and social activities. The fears of patients with LVAD can be categorized into device-related, transplant-related, and psychological/emotional fears. Device-related fears involved maintaining the device in optimal condition, transplant-related fears included not surviving until transplantation or not receiving an organ, and psychological/emotional fears related to sexuality and disease progression, correlating with anxiety and depression. The fears experienced by heart transplant recipients fall into three main categories: avoidance, existential, and psychological fears. Avoidance fears lead to lower exercise motivation and higher anxiety, existential fears involved the fear of death leading to poor psychological well-being, and psychological fears included concerns about non-compliance repercussions, hypochondriacal responses, and appearing ungrateful.

**Summary:**

Each stage of HF disease presents unique fears with distinct implications, emphasizing the need for stage-specific psychological support and interventions. Further studies are required to understand the impact of fears in different stages of HF disease.

**Supplementary Information:**

The online version contains supplementary material available at 10.1007/s11897-025-00705-0.

## Introduction

Heart failure (HF) is a complex, multifactorial syndrome resulting from impaired heart function. Globally, there are 64.34 million cases of HF (8.52 per 1,000 people), resulting in 9.91 million years lived with disability and costing 346.17 billion US dollars [1]. When medical management of patients with HF is no longer effective, mechanical circulatory support with left-ventricular assist device (LVAD) and heart transplantation are the only available options for significantly extending the life for patients with HF [2].

Patients with HF experience various emotional reactions, among them fears. According to a systematic review, fears related to chronic illness belong to the construct of health anxiety. A health anxiety construct provides a cohesive conceptual view on the common fears of illness symptoms worsening or recurring in individuals with chronic diseases [3]. There is a slight overlap between the disease- or symptom-specific health anxiety perspective, consisting of realistic fear, worry, and concerns, and psychiatric perspective of health anxiety, consisting of symptom misinterpretation, excessive thoughts/feelings and avoidance/reassurance seeking [3]. The influence of health anxiety on quality of life is well established, with evidence suggesting that health anxiety leads to: a) reduced adherence to treatment; b) less engagement in positive health behaviors; and c) higher medical costs [3]. In studies involving cardiac patients, the research concentrated on the concept of cardiac/heart focused anxiety, which is defined as a fear of heart-related stimuli and sensations due to their perceived adverse outcomes [3, 4]. Table 1 presents definitions of the concepts used in the review.

**Table 1:** Main concepts’ definitions

Concept	Definition
Health anxiety	The fear and worry that occurs in response to living with a chronic illness (3)
Disease-related fears	Fear and worry occurring in response to living with a chronic illness and is a part of health anxiety (3)
Cardiac/heart focused anxiety	A fear of heart-related stimuli and sensations due to their perceived adverse outcomes (3)
Existential fears	A fear of nonexistence and a sense of isolation from one's environment (5)
Death anxiety/Fear of death	The state in which an individual experiences apprehension, worry, or fear related to death and dying (16)
The fear-avoidance model	A self-perpetuating cycle of anxiety, fear, and avoidance behaviors related to pain that leads to chronic pain development (42)

Understanding how fears may differentially influence patients with HF depending on the stage of the disease, such as advanced HF, following LVAD implantation and following heart transplantation is thus essential for delivering personalized care. The purpose of this review is to outline how the HF disease trajectory is affected by patient’s fears.

## Methods

A literature search was conducted in Google Scholar, PubMed, CINAHL and PsycINFO for studies published between January 2004 and March 2024. The 20-year timeframe was chosen to capture both recent and relevant research while allowing for historical context in the study of fears among heart failure patients. Foundational references predating 2004 were selectively included if they provided essential theoretical models or definitions relevant to understanding fear in chronic illness. The literature search was conducted independently by two authors (SM and MW). Studies were included if they: (a) were published between January 2004 and March 2024; (b) examined emotional or psychological responses—particularly fear—among adults with advanced heart failure, LVAD, or heart transplantation; (c) were peer-reviewed articles in English; and (d) used either quantitative, qualitative, or mixed-method designs. Exclusion criteria included editorials and non-peer-reviewed sources. Screening was performed in two stages: title/abstract screening followed by full-text review. Discrepancies between reviewers were resolved through discussion. Data extraction included authors, year, study type, population, disease stage, type of fear identified, and its psychological/clinical outcomes. A narrative synthesis approach was used to integrate findings across studies. Due to the diversity of study designs, a formal meta-analysis was not feasible.

This narrative review applied a structured approach to data extraction, grouping the findings thematically by disease stage (advanced HF, LVAD, and post-transplantation) and fear type (disease-related, existential, psychological). Studies were analyzed according to their design and key outcomes related to patient fears. This allowed us to compare and contrast findings across qualitative, quantitative, and mixed-methods studies.

## Results

The initial search across four databases (Google Scholar, PubMed, PsycINFO, and CINAHL) yielded 684 articles. After removing duplicates and screening titles and abstracts, 97 full-text articles were reviewed. Of these, 53 studies met the inclusion criteria and were included in the final review. The included studies consisted of 22 quantitative, 19 qualitative, and 3 mixed-method designs. In addition, we reviewed 4 review papers, 4 theoretical articles, and 1 book/manual, covering fears across different stages of HF, including advanced HF, LVAD support, and post-transplantation.

## Prevalence and Implications of Fear in Patients with Advanced Heart Failure

Two main types of fear have been identified among patients with advanced HF: disease-related fears, which include fear and worry in response to living with a chronic illness (often categorized under health anxiety) [3], and existential fears, defined as “a fear of nonexistence and a sense of isolation from one's environment” [5].

Disease-related fears encompass a variety of psychological responses. Fear of disease progression, for example, has been shown to correlate positively with symptom perception and negatively with self-care confidence, mental resilience–strength, and mental resilience–toughness among patients with HF hospitalized in cardiology departments [6]. Fear of disease progression has also been associated with poor sleep quality among hospitalized patients with chronic HF [7]. Physical symptoms, such as dyspnea, are commonly associated with increased fear in hospitalized patients with HF [8]. Another prominent fear is fear of physical activity (FoPA). FoPA significantly and consistently predicted reduced ability to walk up and down stairs independently among outpatients with HF, even after controlling for covariates such as sex, age, body mass index (BMI), living status, employment, participation in cardiac fitness groups, and engagement in other sports activities. Notably, FoPA did not differ by gender [9]. Moreover, FoPA was positively associated with distress related to HF symptoms and with outpatients’ inaccurate perception of their own heartbeats (i.e., lower interoceptive accuracy) [10]. Additional study has found that hospitalized patients with HF who experience depression, sleep disorders, and multiple comorbidities exhibit increased fear of falling, while higher left ventricular ejection fraction (LVEF) is linked to lower fear of falling [11]. Among both outpatient and inpatient patients with stable HF, higher levels of general anxiety and reduced kidney function (measured via estimated glomerular filtration rate, eGFR) were associated with greater heart-focused anxiety, suggesting that both psychological and physiological factors influence fear in this population [12]. Similarly, fear of movement has been associated with several psychosocial factors. A cross-sectional study among patients with HF hospitalized in cardiology units showed that fear of movement was positively associated with cardiac anxiety, depressive symptoms, and employment status, and negatively associated with subjective social status [13]. Finally, a qualitative study exploring treatment compliance among patients hospitalized for symptomatic HF found that fear of worsening symptoms and rehospitalization was a major motivator for adhering to treatment recommendations—such as medication use, fluid and sodium restrictions, and daily weighing. In contrast, barriers to compliance included the poor taste of sodium-restricted food, thirst caused by fluid limits, forgetfulness, and misunderstanding of care instructions [14]. For men with chronic HF interviewed in the outpatient clinic, the first fear they reported upon becoming ill was the possibility of no longer being able to continue working [15]. Taken together, disease-related fears in advanced HF are linked to reduced psychological resilience, increased emotional distress (e.g., anxiety, depression), decreased physical and professional functioning, and variable treatment adherence, underscoring the need for individualized psychological and educational interventions.

Among patients with advanced HF, existential fears included fear of death. Fear of death or death anxiety has been defined as “the state in which an individual experiences apprehension, worry, or fear related to death and dying” [16]. Among outpatients with advanced HF, the fear of death was described as"living in the shadow of fear. This fear predominantly accompanied individuals at night, especially during episodes of nocturnal breathlessness [17]. At the end of life, both inpatients and outpatients with HF often experience fear, particularly when suffering from severe shortness of breath [18]. An earlier study found that elderly outpatients with HF experienced significant contemplation about death, with higher levels of anxiety and depression correlating with increased fear of mortality [19]. Among women with HF who were outpatients in an HF clinic, fear of death during clinical symptoms such as palpitations, along with fear of living with a frail and failing body and loss of dignity, contributed to feelings of insecurity in the present [20]. Fear of dying has been reported in a qualitative study by 32.8% of participants who were outpatients with chronic HF, as a mental health sequelae with a day-to-day impact [21]. The heartache of fear associated with uncertainty—along with thoughts of mortality, lifespan, and prognosis—affected the confidence of outpatients with chronic heart failure to engage in daily activities, including physical exercise and hobbies they once enjoyed [22]. Fears of being a burden to the caregiver, breathlessness-related fears, and fear of dying were common among outpatients with heart failure, often leading to sleeplessness and anxiety [23]. Similarly, fears of being a burden to family and friends led outpatients with HF to avoid or deny their symptoms, consequently preventing them from seeking treatment [24]. Feelings of abandonment and a fear of being left alone characterized severe patients with chronic HF in palliative advanced home care [25]. A narrative review of sixteen qualitative studies, most of which were previously described, found that fear of death, pain, or uncertainty about the future emerged as central to the lived experience of patients with chronic heart failure. The review demonstrated that patients restricted their physical and social activities due to fears about personal safety and their perceived inability to manage or control situations [26]. During the COVID-19 pandemic, a high prevalence of fear of COVID-19 among patients with HF has been reported, with psychological concerns arising from the debilitating impact of the virus [27] leading HF patients avoiding urgent medical care [28, 29].

Across the continuum of advanced heart failure, fears manifest differently depending on patients’ care settings. Hospitalized patients with advanced HF tend to report heightened fears related to disease progression, worsening symptoms such as dyspnea, rehospitalization, and fears of falling and movement. In contrast, community-dwelling patients with advanced HF often experience fears embedded in their daily routines and social environments, including fear of physical activity, and fear of losing the ability to work. Existential fears—such as fear of death, burdening others, and isolation—are also particularly prevalent in home-based settings. Recognizing these context-specific differences is essential for tailoring psychological and supportive care interventions across care settings. As can be seen, disease-related and existential fears, frequently accompanied by shortness of breath, were associated with anxiety, depression, sleeplessness, loss of dignity, feelings of abandonment and uncertainty about the future, and were sometimes linked to restricted physical and social activities.

## Prevalence and Implications of Fear in Patients with LVAD

The fears of patients with LVAD can be categorized into three main categories: device-related, transplant-related and psychological and emotional fears. Device-related fears included concerns that arose during the decision-making process among patients with advanced HF who were offered an LVAD. These fears included concerns about the surgery, exacerbation of pre-existing conditions, and engaging in activities such as cooking, gardening, and having sex after being implanted with an LVAD [30]. The fear of death often dominates the decision-making process for patients with advanced HF considering a destination therapy LVAD [31, 32]. Following LVAD implantation, the device-related fears included concerns of device malfunction and complications [33, 34]. The participants feared that the device would malfunction due to several reasons. The reasons could be the fear of the device breaking down due to being cursed, jamming, or breaking when handled. Moreover, the patients were describing fear of complications that would arise, such as fear of possible infection, thrombosis, or bleeding. Another fear was the uncertainty which was expressed as fear of the device being stolen. As the device is carried in a bag it could potentially be snatched [33]. Patients with LVAD expressed fear related to LVAD (ie. alarms and disconnecting driveline) during intimate relationships [35]. In a qualitative phenomenological study, patients described that living with LVAD made them adjust their daily routine, causing fear of making mistakes, particularly with the handling of the device. This included fear of breaking sterile technique or making errors when handling the device [36]. To conclude, device-related fears of patients with LVAD were associated with patients’ attempts to keep and maintain the device in optimal condition.

Transplant-related fears included transplantation uncertainty and isolation. Carrying an LVAD resulted in fear of not receiving an organ, also a fear of being left alone since many are dependent on support from family members to handle the maintenance of the LVAD [33]. In a mixed-method study, patients described a fear of not surviving until the time of transplantation. The fear was related to the decision to wear an LVAD while waiting for a heart transplant could potentially cause the patient to slip down the waiting list. Furthermore, wearing an LVAD was a reminder of a worsening prognosis, amplifying the fear of not surviving until the time for transplantation [37]. There were also patients that for some reason cannot receive a heart transplant and must rely on LVAD as a permanent solution. In a thematic interview study with patients who were deemed ineligible for heart transplant, they expressed fear knowing that access to trained medical professionals may be limited in case of emergencies related to LVAD [38]. To conclude, transplant related fears of patients with LVAD mainly included the fear of not surviving until the time of heart transplantation or not receiving an organ.

Psychological and emotional fears included fear related to sexuality, as reported in a cross-sectional study where patients with LVAD reported sexual dysfunction due to fear of injury. The fear of injury in relation to sexuality were more prominent in patients with LVAD than after the transplant [39]. One of the concerns among patients with LVAD was the fear of disease progression. Patients with LVAD often experienced anxiety about the progression of their condition and its impact on their health and treatment [40]. A cross-sectional study aimed to gain understanding of couples after LVAD implantation found that patients with LVAD had a strong fear of progression and that the fear was correlated to depression and anxiety. The same study reported that caregivers experienced the same fear of progression and, in some circumstances, even more distress than the patient, possibly increasing the burden of distress and fear for the patient with an LVAD [40]. Among couples where one spouse had an LVAD implanted, the fear theme was based on previous traumatic events before or during LVAD implantation, pain and difficulties following the operation, and life dependence on electricity [41]. To summarize, the psychological and emotional fears of patients with LVAD included fears related to disease progression, pain and difficulties following the operation, and life dependence on electricity and correlated with anxiety and depression.

## Prevalence and Implications of Fear in Patients Post Heart Transplantation (HTx)

The fears experienced by heart transplant recipients can be categorized into three main categories: fear-avoidance beliefs and behaviors [42], existential, and psychological fears. The fear-avoidance model, proposed by Lethem et al. [42] suggests that chronic pain develops through a self-perpetuating cycle of anxiety, fear, and avoidance behaviors related to pain. In the case of HF patients, fears were associated with avoidance beliefs and behavior. Avoidance beliefs characterized patients with LVAD who feared the surgical risks associated with accepting a heart transplant after already undergoing LVAD implantation surgery [43, 44]. Patients after HTx experienced a fear associated with avoidance—a fear of movement (kinesiophobia). Kinesiophobia is characterized by an extreme, unfounded, and incapacitating fear of physical movement and activity, stemming from a perceived susceptibility to pain or further injury [45]. A cross-sectional study among patients after HTx found that time since transplantation, total exercise self-efficacy, and extrinsic motivation were negatively correlated, while the total disability score was positively correlated with kinesiophobia [46]. Additionally, a qualitative study employing a phenomenological-hermeneutic method highlighted that heart transplant recipients often lived in a state of uncertainty, including fears related to medication complications (e.g., cancer, diabetes, or kidney failure) and concerns about infection risks during recovery [47]. While these fears were not explicitly categorized as avoidance-related, they reflect emotional distress that may influence patients'behavior and sense of security post-transplant [47]. To summarize, fears associated with avoidance among patients after HTx were associated with lower exercise motivation, higher disability, feeling insecure and anxious about recovery.

The second type of fear found among heart transplant recipients was existential fear, which is the fear of death [48, 49]. Moreover, heart transplant recipients reported experiencing fear of graft rejection, with some of them feeling that the risk of graft rejection was out of their control, highlighting another significant existential fear, being associated with poor psychological well-being [50].

**Fig. 1:**
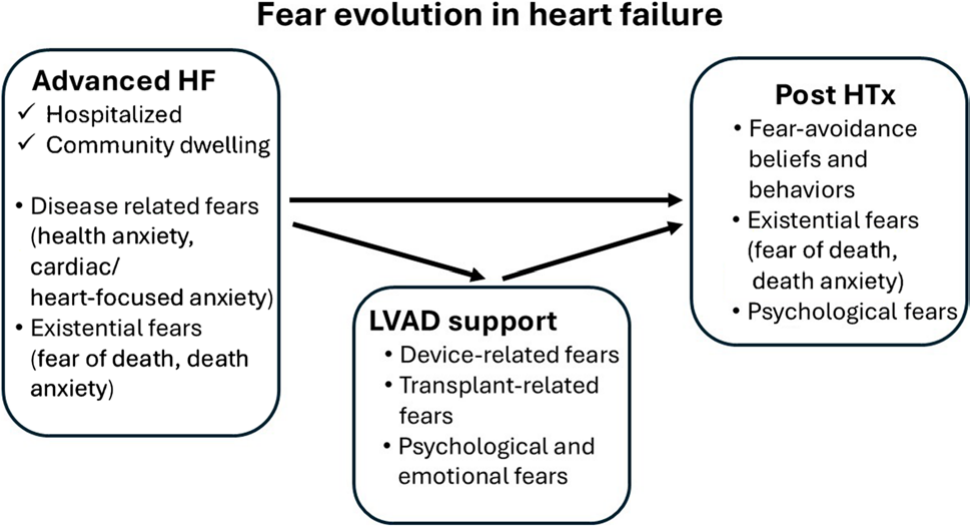
The prevalence and nature of fear in different stages of heart failure disease

The third type of fear among heart transplant recipients is psychological fear. Previous phenomenological study found heart transplant recipients felt contradictory paradoxical emotions, among them “fear versus carelessness” or fear of repercussions from non-compliance: After returning to what felt like normal life post-transplant, participants sometimes ignored medical advice, driven by a psychological fear of facing repercussions for these actions in the next 10 to 15 years [51]. The research comparing heart transplant recipients to matched controls revealed a significantly higher occurrence of at least one"hypochondriacal response"among the transplant patients. The majority of these responses were related to the disease phobia scale, exploring psychological fears, with patients'fears being related to heart disease, cancer, and other severe illnesses [52]. In addition, it's not unusual for heart transplant recipients to experience intense feelings of gratitude to those around simultaneously mixed with anger, survivor's guilt, and the fear of seeming ungrateful [53]. To conclude, psychological fears of transplant patients included fears of non-compliance repercussions, hypochondriacal responses concerning severe illnesses, and fear of appearing ungrateful. Figure 1 presents schematic representation of the main study concepts.

## Discussion

The findings of this review highlight the complex and evolving nature of fear among patients across the HF trajectory, including advanced HF, LVAD support, and post-heart transplantation. Fear is a multifaceted emotional experience that can significantly impact psychological well-being, treatment adherence, and quality of life in this patient population.

### Evolvement of fear across the heart failure trajectory

Fear in patients with advanced HF is predominantly characterized by disease-related and existential fears. These include fear of worsening symptoms, fear of physical activity, and fear of death. Such fears are often triggered by symptoms like dyspnea and the unpredictability of the disease course. As patients transition to LVAD support, fear shifts toward device-related concerns (e.g., fear of mechanical failure or infection), transplant-related fears (e.g., not receiving an organ), and psychological fears (e.g., loss of independence and intimacy). Following transplantation, patients experience new fears, including fear avoidance beliefs and behaviors like kinesiophobia and physical movement and activity avoidance, and existential concerns such as fear of death and of graft rejection, and fear of repercussions from non-compliance as well as fear of being perceived as ungrateful. This progression illustrates how the focus of fear changes in accordance with the clinical realities and psychological demands of each HF stage.

### Integration with theoretical frameworks

The empirical findings align with established theoretical models such as health anxiety and the fear-avoidance model. Health anxiety encompasses realistic fears and concerns about illness progression, as well as excessive worry and misinterpretation of bodily sensations. The fear-avoidance model helps explain how fear can lead to behavioral avoidance, as seen in patients'reluctance to engage in physical activity or adhere to treatment regimens. Both frameworks provide valuable lenses through which to understand patient behaviors and emotional responses throughout the HF journey.

### Conceptualization of fear in a health context

This review proposes a three-dimensional conceptualization of fear in HF: disease-related, existential, and psychological. Disease-related fear pertains to physical symptoms and disease progression. Existential fear reflects concerns about mortality and the meaning of life. Psychological fear includes fears of non-compliance repercussions, loss of identity, and interpersonal issues. Recognizing these dimensions is essential for comprehensive patient assessment and personalized care planning.

### Implications for HF management and the role of family

Clinicians must acknowledge fear as a significant factor in HF management. Early screening and intervention for fear-related distress can improve adherence, reduce hospitalizations, and enhance patient well-being. Psychological support should be tailored to the patient’s disease stage, with family involvement encouraged where appropriate. For example, caregivers play a vital role in managing device-related fears in LVAD patients and offering emotional support during transplantation recovery. Education, counseling, and reassurance can help mitigate fears, improve coping mechanisms and resilience.

### Research gaps and future directions

There is a need for longitudinal studies to explore how fear evolves over time and in response to treatment milestones. Research should also address cultural, gender, and age-related differences in the experience of fear. Further investigation into interventions targeting specific types of fear and their impact on clinical outcomes is warranted. Additionally, integrating qualitative and quantitative approaches can deepen our understanding of patient experiences and inform holistic care strategies.

In summary, fear is a dynamic and pervasive aspect of living with HF. Addressing its presence across the disease continuum requires stage-specific, theory-informed, and person-centered approaches to care.

## Limitations

This review has several limitations. First, the search terms primarily focused on 'fear' in heart failure patients. While this targeted approach allowed for a focused analysis, it may have excluded studies addressing broader emotional responses such as health anxiety, emotional distress, or coping mechanisms. Future reviews could expand the keyword strategy to capture a wider range of psychological experiences across the trajectory of heart failure.

Second, while both qualitative and quantitative studies were reviewed, no formal triangulation technique was applied. Future research could benefit from using mixed-method synthesis frameworks to further validate and enrich understanding of patients'fears across disease stages.

Despite these limitations, this review provides valuable insights into the prevalence and implications of fears in HF patients, highlighting the need for stage-specific psychological interventions and further research in this area.

## Conclusion

This review underlines the central role of fear in the experience of patients with HF across the disease trajectory. Fear is not a monolithic experience but transforms in response to changing clinical conditions—from physical symptom-related fear in advanced HF, to technology- and prognosis-related concerns during LVAD support, to existential and psychological fears after heart transplantation. The fear in the context of HF is multifaceted: it encompasses disease-related anxieties, existential distress, and fears rooted in self-perception, autonomy, and interpersonal relationships. These fears impact not only emotional well-being but also physical functioning, self-care adherence, and quality of life.

Clinical management of HF should therefore recognize fear as a dynamic psychological factor. Tailored interventions—timed and targeted to patients’ disease stage—should aim to reduce maladaptive fear responses, support emotional coping, and empower patients in self-care. Moreover, the involvement of family members and healthcare professionals into these processes is critical, as they serve as both potential sources of encouragement and unintended contributors to distress.

Future research must move beyond cross-sectional snapshots and begin to chart the temporal progression and contextual variability of fear in HF, with particular attention to cultural and gender-related dimensions.

## Supplementary Information

Below is the link to the electronic supplementary material.

Supplementary file1 (PDF 1.33 MB)

Supplementary file2 (PDF 139 KB)

## Data Availability

No datasets were generated or analysed during the current study.
